# The Etiology of Empyema

**Published:** 1903-01-17

**Authors:** J. Odery Symes

**Affiliations:** Assistant Physician, Bristol General Hospital.


### HOSPITAL CLINICS AND MEDICAL PROGRESS.

### THE ETIOLOGY OF EMPYEMA.

By J. ODERY SYMES, M.D., D P.H., Assistant Physician, Bristol General Hospital.

DURING the past few years I have had the opportunity of making a bacteriological examination of the exudate in twenty-four cases of empyema. Of these twenty four, twelve showed the presence of pneumococci, eight of streptococci, three of staphylococci, and one was sterile.

Dealing with the cases of pneumococcus infection first, we find that in ten the organism was found alone, in three it was associated with the staphylococcus, in one with putrefactive bacteria, and in one with staphylococci and bacilli. Pure pneumococcus infection is generally acknowledged to warrant a more favourable prognosis than that by any other organism, and it has been maintained, more especially by French observers, that this condition may be cured by aspiration alone without subsequent incision and drainage of the pleural cavity. Whether this be so or not, there can be no doubt that pneumococcus empyemata are more amenable to treatment than any others, and it is noteworthy that in the present series seven out of the ten pure pneumococcus infections occurred in children. The finding of pneumococci together with putrefactive organisms in the pus of an empyema is of some diagnostic value as it would point to the presence of gangrene of the lung or to the rupture of an abscess or ulceration of a cavity into the pleura. If putrefactive organisms are present, and especially if these be of the coli type, and pneumococci are found, the pleura has probably been invaded by an abscess in connection with the abdominal viscera, or the case may be one of subphrenic abscess. The prognosis as regards the ultimate closure of the cavity is unfavourable in cases of foul smelling empyemata, and these accompanied by formation of gas.

Of the eight streptococcal cases, the organisms were found alone in two, with pneumococci in two, with staphylococci and B. influenzæ in one, with staphylococci and tubercle bacilli in one, with putrefactive bacilli in one, and with pneumococci and B. termo in one.

Six out of the eight streptococcal empyemata, including the two pure infections, occurred in adults. In the case in which the streptococcus was found along with the pneumococcus and B. termo the pus was offensive and of a green colour. This case terminated fatally, as also did that in which putrefactive organisms occurred. It has not been possible to follow out the history of all of the cases in this series, but my notes show that most of the deaths recorded occured in the streptococcus group. Undoubtedly the prognosis in streptococcus empyema is very grave.

The three staphylococcus infections all occurred in adults. Not infrequently the empyema is a secondary pyæmic infection, as in one case which developed during the treatment of a bad whitlow of the finger. As a rule staphylococcus empyemata do not do well, very probably because in many cases they are complicated by tubercle. It is seldom possible to demonstrate the presence of tubercle bacilli in the exudate by the ordinary methods of staining, nor is it possible to obtain cultures. Should there be any suspicion that the condition is tubercular, then the pus should be submitted for testing by inoculation. In the last case of this series in which the pus was sterile it is possible that the disease was tubercular. If the pus from an empyema show no organisms, or if tubercle bacilli be found, then the question of incision and drainage will have to be reconsidered, for in tubercular cases we do not see favourable results following these operative procedures such as we do when other organisms are present. Occasionally there is a difficulty in identifying the pneumococcus, and this can only be finally settled by inoculation. It should be remembered that the pneumococcus is pleomorphic, and in stained films of the pus may be found not only as the encapsuled lanceolate diplococcus but in short chains as a streptococcus, or in diplo- or rarely staphylococcus form. Occasionally in empyemata of old standing it will be found that cultures made from the pus are sterile, and that direct films from the exudate show only partially stained organisms, the nature of which it is impossible to discover. In such cases we may presume that the organisms have died, and there is the possibility that if left alone the fluid contents may in part become absorbed and the remainder undergo caseation. The nature and variety of the pus found in empyemata vary with the organisms present. Thus, when the pneumococcus is the infective agent the pus is greenish, thick and creamy; with the tubercle bacillus thin and flakey; with the bacillus coli, B termo, and other putrefactive varieties, offensive and perhaps accompanied by the formation of gas. Once the chest has been incised and drained, however carefully the operation may have been performed, the bacteriological contents of the pus undergo alteration and staphylococci are almost always introduced. The pus to be examined should be that drawn off by the initial exploratory puncture, and direct films should be spread from this and stained with methylene blue, and by Gram's and Liebb Neelsen's methods. Cultures are best made on blood agar or suspessated blood serum. A small portion of the pus drawn off should be retained in order that it may be used for inoculation purposes should there be any doubt as to the result of the microscopical and cultural examinations.

